# Association between ensuring a daily rest period in the workplace and workers’ sleep: Evidence from a matched employer–employee survey in Japan

**DOI:** 10.1016/j.ssmph.2026.101967

**Published:** 2026-09-14

**Authors:** Huilin Wang, Hideyuki Kobayashi, Masaya Takahashi

**Affiliations:** a Social Science and Occupational Health Research Group, National Institute of Occupational Safety and Health, Japan; b Institute for Research on Community Health, Well-Being and Coexistence, University of Kochi, Japan; c Research Center for Overwork-Related Disorders, National Institute of Occupational Safety and Health, Japan

**Keywords:** Daily rest period, Work-interval system, Weekly working hours, Overtime hours, Short sleep duration, Sleep problems

## Abstract

**Objectives:**

This study examined how ensuring a daily rest period (DRP) in the workplace was associated with workers’ sleep and whether workplace-level DRPs moderated the association between working hours and sleep, with the exploratory aim of identifying potential differences based on gender.

**Methods:**

Using cross-sectional data from a matched employer**–**employee survey, this study analyzed a sample consisting of 4202 full-time workers from 2121 workplaces. Logistic regression models were employed to predict sleep quantity and quality outcomes.

**Results:**

There was no significant association between ensuring a DRP in the workplace and short sleep duration among full-time workers. Increasing working hours was significantly associated with higher odds of short sleep duration; however, this association appeared weaker among workers in workplaces with ≥11 h DRP. Exploratory analyses stratified by gender suggested that this pattern appeared more evident among women than among men. Moreover, ensuring ≥11 h DRP was significantly associated with lower odds of short sleep duration among overtime workers and daytime sleepiness among full-time workers. Gender-specific differences in insufficient sleep, early morning awakening, daytime sleepiness, and nonrestorative sleep were observed.

**Conclusions:**

The findings suggest the relevance of introducing a work-interval system or taking measures to ensure a DRP in the workplace for workers’ sleep health. Nevertheless, further longitudinal, interventional, or quasi-experimental studies are required to establish the causal relationship between ensuring a DRP and sleep outcomes.

## Highlights

• Workplace-level daily rest period (DRP) and workers' sleep were examined.
• Increasing working hours was associated with higher odds of short sleep.
• The association was weaker for workers with ≥11 h DRP, especially for women.
• DRP≥11 h was associated with lower odds of short sleep among overtime workers.
• DRP≥11 h was associated with lower odds of daytime sleepiness.

## 1 Introduction

Sleep is a fundamental physiological activity and a crucial pillar of health and well-being (Duncan et al., 2019). Epidemiological and economic evidence consistently highlights the health and economic consequences of poor sleep. A large body of literature documents that short sleep duration is associated with higher risks of various adverse health outcomes, such as poor cognitive function, cardiovascular disease, coronary heart disease, and mortality (Cappuccio et al., 2010; Itani et al., 2017; Lo et al., 2016). Additionally, sleep problems have been shown to be associated with a range of health conditions, including incident cardiovascular disease (Bertisch et al., 2018), depression (Baglioni et al., 2011), disability retirement (Lallukka et al., 2011), suicide, and overall mortality (Rod et al., 2011). Hence, it is widely recognized that good sleep is essential for good health (Buysse, 2014). Furthermore, poor sleep is associated with reduced workplace performance and productivity (Ishibashi & Shimura, 2020; Rosekind et al., 2010). In Japan, the annual economic costs of insufficient sleep are estimated to be as high as 2.9% of the country's GDP (Hafner et al., 2017).

The high prevalence of short sleep duration among the working population in Japan has long been in the spotlight. According to a recent nationally representative survey, 45.5% of workers reported sleeping for fewer than 6 h a day and 69.3% claimed that they could not obtain the amount of sleep they desired (Ministry of Health, Labour and Welfare, 2023), implying serious chronic sleep deficiencies, which need to be addressed.

Although a broad range of work-related factors have been identified as inducing poor sleep, long working hours are considered the most relevant contributor (Bannai & Tamakoshi, 2014; Wong et al., 2019) because they directly reduce total sleep time (Dahlgren et al., 2006; Wang et al., 2026). The prevalence of long working hours remains high worldwide. Approximately one-third (36.1%) of the global workforce works for more than 48 h per week (Messenger, 2018). Furthermore, overwork culture is deep-rooted in Japan, where karoshi (overwork-related death) has been continually increasing and remains a major public health concern (Takahashi, 2019). To prevent karoshi and promote work-life balance, health, and well-being among workers, Japan introduced a series of new regulations in the 2019 Work Style Reform, including the implementation of a work-interval system in the workplace and mandatory limits on overtime work (Burdin et al., 2024). The work-interval system, which ensures a certain number of rest hours between the end of work on one day and the start of work on the next day, is similar to the daily rest period (DRP) introduced in European countries (Takahashi, 2022). The prevalence of short DRPs among workers is high. For instance, a previous study conducted in Japan reported that 54.5% of employees had less than 11 h DRP at least once a month (Kubo et al., 2018). Therefore, implementing the work-interval system is expected to ensure sleep opportunities and, in turn, promote workers’ health and well-being.

Empirical evidence has shown that a shorter DRP was significantly associated with shorter sleep duration, greater sleep debt, social jetlag, and an increased risk of sleep problems (Ikeda et al., 2018, 2021). By contrast, a longer DRP was associated with improved sleep quantity and quality (Ikeda et al., 2022). These studies used a recall method by asking workers their usual end and start times of work over the past month, and defined DRP as the interval between workers’ self-reported end and start times of work (Ikeda et al., 2021, 2022). DRP defined in this way reflects an average level over the past month. Consequently, workers might experience short DRPs on specific days, which may not be captured by the monthly average. Moreover, as working hours are not accounted for, the estimated association between DRPs and sleep may be confounded by working hours.

Another strand of literature has relied on quick returns (defined as <11 h DRP) and has focused primarily on the healthcare sector. Evidence from Norwegian nurses has shown that quick returns were associated with insomnia, excessive sleepiness, excessive fatigue, and shift work disorder (Eldevik et al., 2013). A longitudinal study found that quick returns at baseline increased the risk of shift work disorder and pathological fatigue at 1-year follow-up, whereas reducing the number of quick returns from baseline to follow-up was associated with reduced risk of pathological fatigue (Flo et al., 2014). A study of Swedish nurses using objective sleep data collected over seven consecutive days reported that quick returns were associated with 1 h shorter sleep length, reduced subjective sleep quality, increased anxiety at bedtime, and increased worktime sleepiness (Öster et al., 2024). However, research should further investigate whether workplace implementation of reducing quick returns may lessen sleep complaints among workers (Eldevik et al., 2013).

To date, no evidence exists regarding how ensuring a DRP as a workplace implementation is associated with workers’ sleep. To fill this research gap, we used matched employer–employee survey data and hypothesized that ensuring a DRP in the workplace would be associated with reduced risks of sleep problems.

Short DRPs are usually induced by long working hours or demanding work schedules. In certain sectors, such as healthcare, transportation, and services, the prevalence of demanding work schedules is high (Harknett et al., 2020). An evening schedule followed by a day schedule or a night schedule followed by an evening schedule may result in short DRPs. For daytime workers, extending working hours combined with an early or normal start time on the next day may result in short DRPs. In workplaces with a work-interval system, workers who work overtime can start work later the next day (e.g., 10 a.m. instead of 8 a.m.) to have a sufficient DRP, as shown in Fig. 1. Instead of postponing the start time of the next day, some workplaces implement enforced clock-out by automatically shutting down computers or switching off office lights to discourage overtime work and ensure a sufficient DRP. In this study, we focus on full-time workers (35 h or more per week), who are more likely to encounter restricted sleep opportunities due to work commitments. In addition, the relationship between working hours and sleep may vary for workers in workplaces with different DRP implementations. To the best of our knowledge, no study has investigated this issue. Therefore, we investigated whether the relationship between working hours and sleep depends on workplace-level DRP. We hypothesized that ensuring a DRP in the workplace would moderate the relationship between working hours and sleep.

**Fig. 1:**
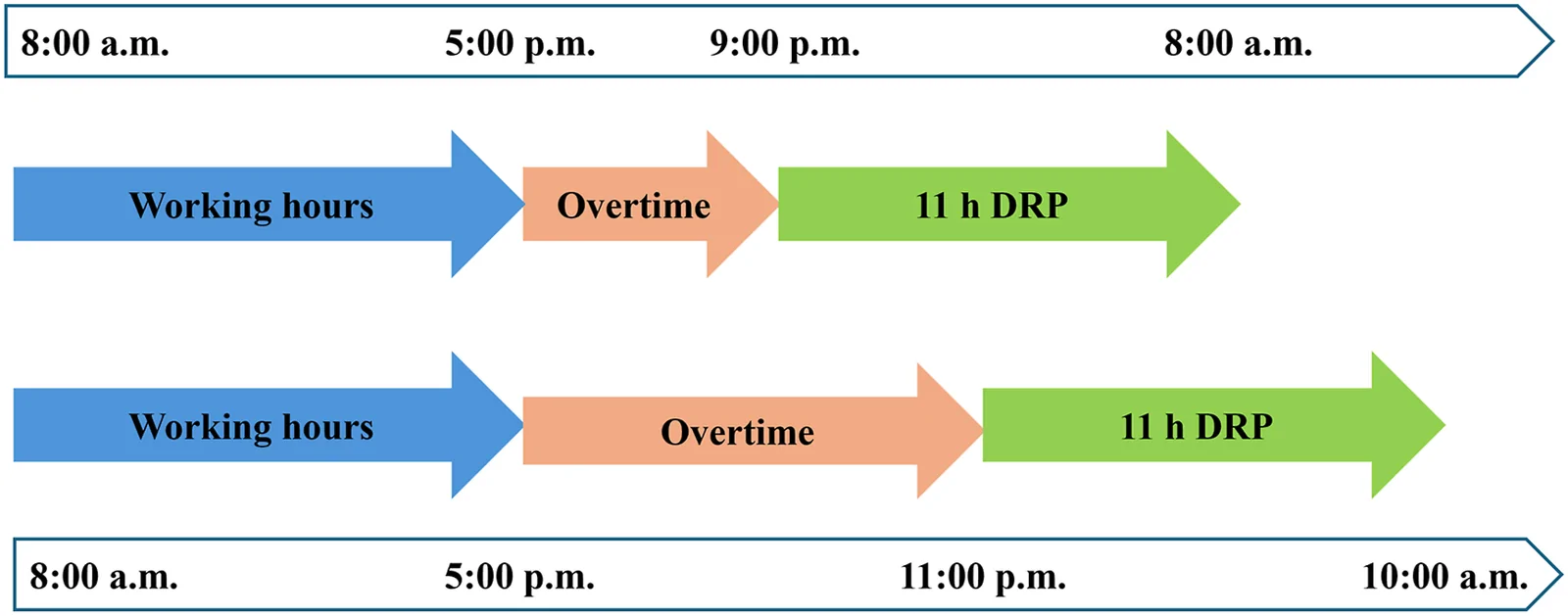
The work-interval system. Note: Working hours include the 8 h statutory working hours and a 1 h lunch break.

Furthermore, previous research has underscored the burden of unpaid work on sleep (Morimoto et al., 2026). In Japan, although women devote less time to paid work than men, they undertake more domestic duties, also called “the second shift.” Profound gender gaps in the division of unpaid work remain a central issue. Japanese men spend the least amount of time on unpaid work among OECD countries, averaging only 47 min per day, which is substantially lower compared to other countries, such as 139 min in Australia, 150 min in Germany, 140 min in the UK, and 145 min in the US (OECD, 2024). Women with short DRPs may have to shorten sleep to perform domestic duties. Therefore, ensuring a DRP may play an important role in securing sleep opportunities for women. Currently, there is a lack of evidence regarding whether the association between DRPs and sleep differs by gender. To address this gap, we conducted subgroup analyses stratified by gender to explore potential gender differences and hypothesized that the association between DRPs and sleep would be stronger among women than among men.

## 2 Methods

### 2.1 Data

This study utilized cross-sectional data from a matched employer**–**employee health and work-life survey conducted between December 2022 and February 2023. To obtain a representative sample, we randomly selected 20,000 workplaces based on the sector and scale composition of private workplaces in Japan. Each workplace received one employer and three employee questionnaires. The employer questionnaire was intended to be completed by the safety and health supervisors or general affairs managers at the workplace. The employee questionnaires were randomly distributed. Separate envelopes were included so that employees could mail back their completed questionnaires without worrying that their responses would be disclosed at the workplace. Ethical approval was obtained from the Research Ethics Committee of the National Institute of Occupational Safety and Health, Japan (No. 2022N23). Respondents were informed that participation in the survey was voluntary, and that the data would remain anonymous, impersonal, and would be used only for scientific purposes. A total of 3103 workplaces and 6449 employees participated in the survey. The advantage of our dataset is that it contains detailed workplace-level information and rich information on employees' job characteristics and health conditions. The derivation of the study sample is shown in Fig. 2. The final sample for analysis consisted of 4202 employees from 2121 workplaces.

**Fig. 2:**
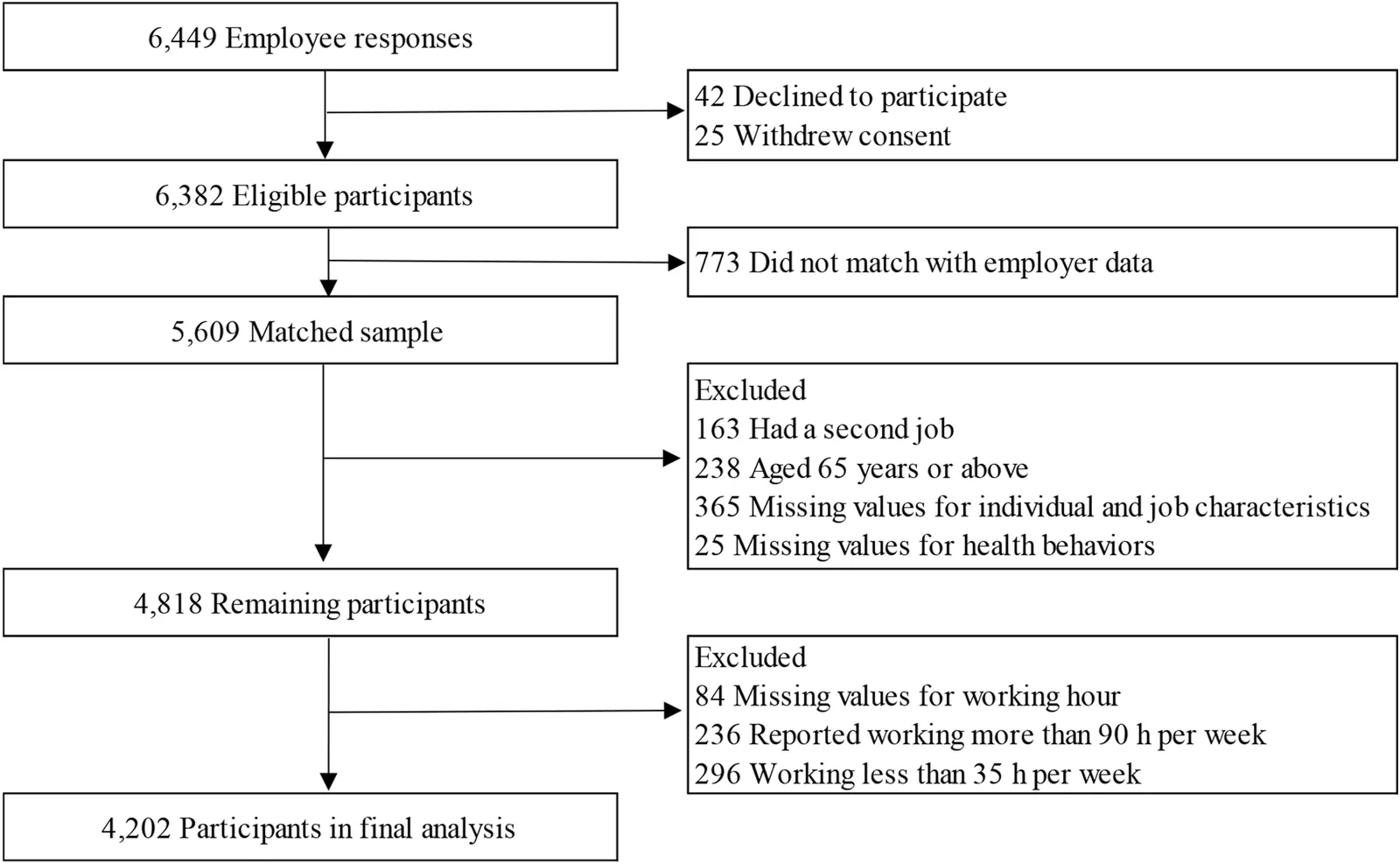
Derivation of the study sample.

### 2.2 Variables

#### 2.2.1 DRP in the workplace

In contrast to previous research using workers’ self-reported DRPs, this study used a workplace-level DRP, that is an implementation in the workplace aimed at ensuring a minimum DRP for all employees. In the employer survey, safety and health supervisors or general affairs managers at the workplace were asked whether the workplace had adopted measures to ensure a DRP for employees. The options were as follows: (1) has a work-interval system formally established in a collective agreement, labor-management agreement, or employment rules; (2) has not formally introduced a work-interval system but has taken measures to ensure a DRP; (3) no measures at present but considering introducing measures to ensure a DRP in the future; (4) no measures at present and not considering introducing any measures to ensure a DRP in the future; and (5) completely unaware of any relevant implementation for ensuring a DRP. Those who selected options (3)–(5) were classified as “no measures.” For those who selected options (1) and (2), we inquired further regarding the number of hours. The options were as follows: <9 h, 9 h, 10 h, 11 h, 12 h, ≥13 h. The European Union Working Time Directive suggests a minimum DRP of 11 consecutive hours every 24 h (European Parliament and Council, 2003), and previous research investigating DRPs or quick returns has generally used 11 h as the cutoff (Eldevik et al., 2013; Flo et al., 2014; Vedaa et al., 2017). Therefore, we selected 11 h as the cutoff and defined a three-category variable: no measures (as reference), <11 h DRP, and ≥11 h DRP. We also defined a variable using 12 h as the cutoff; the estimation results are provided in the Supplemental Material (Table S3 and S4).

#### 2.2.2 Weekly working hours

Weekly working hours were obtained in the employee survey by asking workers: “In the past month, how many hours did you work per week on average?” Our main analysis focused on full-time workers. We also conducted a subsample analysis among overtime workers. In Japan, in principle, statutory working hours must not exceed 8 h per day or 40 h per week. Accordingly, overtime hours were defined as hours exceeding 40 h and were calculated by subtracting 40 h from the weekly working hours. In the analysis, we demeaned weekly working hours and overtime hours by their sample means, such that positive values indicated above-average weekly working hours and overtime hours.

#### 2.2.3 Sleep quantity and quality

Sleep duration was self-reported using the following question: “During the past month, on average, how many hours of sleep did you have?” The response options were as follows: <5 h, 5 h, 6 h, 7 h, 8 h, and ≥9 h. Short sleep duration was defined as less than 6 h. In the survey, we collected a broad range of information regarding sleep, including respondents' desired sleep duration and various aspects of sleep problems. Hence, we also estimated the association of ensuring a DRP with these sleep outcomes. Insufficient sleep was scored as 1 if the respondent's actual sleep duration was less than the desired duration, similar to Ursin et al. (2005). Difficulty initiating sleep was defined as 1 if respondents took more than 30 min to fall asleep (Nakata et al., 2005). Difficulty maintaining sleep, early morning awakening, daytime sleepiness, and nonrestorative sleep were coded as 1 if respondents experienced these sleep problems three or more times per week (Takahashi et al., 2008). Finally, feelings of not recovering from fatigue were defined as 1 if respondents often or always felt they were not recovering from fatigue (Skinner & Dorrian, 2015; Takahashi et al., 2012).

#### 2.2.4 Covariates

For individual characteristics, we controlled for age, gender, educational attainment (coded as 1 if the respondent had a university or higher degree, and 0 otherwise), marital status (coded as 1 if the respondent had a spouse, and 0 otherwise), family size, and health behaviors (physical activity, drinking, and smoking). The options for family size were 1, 2, 3, 4 or more persons. Family size was included as a continuous variable. Physical activity was coded as 1 if the respondent exercised every week. Drinking was coded as 1 if the respondent drank alcohol once or more per week. Smoking was coded as 1 if the respondent was a current smoker. Regarding job characteristics, we controlled for tenure (year), shift work (coded as 1 if yes, and 0 otherwise), and occupation (6 categories).

### 2.3 Statistical analysis

Logistic regression models were employed to estimate the association between ensuring a DRP in the workplace and workers’ sleep. We first estimated a crude model and then a fully adjusted model that included all individual and job characteristics. Next, we added interaction terms between DRPs and working hours and conducted a likelihood ratio test to compare the model with and without interaction terms. To explore potential gender differences, we further conducted subgroup analyses stratified by gender. In all regressions, robust standard errors were clustered at the workplace level to account for correlations among employees in the same workplace. All statistical analyses were performed using STATA 17.

## 3 Results

Table 1 presents respondent characteristics. Overall, 14.1% and 20.5% of respondents worked in workplaces with <11 h DRP and ≥11 h DRP, respectively. Moreover, 51.4% of the respondents were women, and the proportion of women in workplaces with a DRP was lower than that of workplaces with no DRP. In workplaces with ≥11 h DRP, the proportions of clerks and managers were lower, and those of professionals/technicians and service workers were higher than in workplaces with no measures or <11 h DRP. Weekly working hours were highest in workplaces with <11 h DRP.

**Table 1:** Descriptive statistics.

	Full sample		No measures		DRP<11 h		DRP≥11 h	
	N = 4202		N = 2749		N = 592		N = 861	
	n	%	n	%	n	%	n	%
**Age**								
∼29	528	12.6	362	13.2	73	12.3	93	10.8
30∼39	949	22.6	630	22.9	116	19.6	203	23.6
40∼49	1394	33.2	890	32.4	214	36.1	290	33.7
50∼59	1054	25.1	695	25.3	141	23.8	218	25.3
60∼64	277	6.6	172	6.3	48	8.1	57	6.6
Women	2160	51.4	1463	53.2	273	46.1	424	49.2
University or higher	1378	32.8	931	33.9	187	31.6	260	30.2
Married	2463	58.6	1605	58.4	339	57.3	519	60.3
Physical activity	1591	37.9	1019	37.1	231	39.0	341	39.6
Drinking	2213	52.7	1432	52.1	324	54.7	457	53.1
Smoking	925	22.0	570	20.7	158	26.7	197	22.9
Shift work	100	2.4	55	2.0	5	0.8	40	4.6
**Occupation**								
Clerks	1529	36.4	1043	37.9	211	35.6	275	31.9
Managers	389	9.3	257	9.3	62	10.5	70	8.1
Professionals/technicians	697	16.6	444	16.2	93	15.7	160	18.6
Sales workers	315	7.5	212	7.7	38	6.4	65	7.5
Service workers	469	11.2	298	10.8	55	9.3	116	13.5
Manual workers	803	19.1	495	18.0	133	22.5	175	20.3

	Mean	SD	Mean	SD	Mean	SD	Mean	SD

Weekly working hours	43.1	6.1	42.9	5.9	44.1	7.2	42.9	5.9
Family size	2.8	1.1	2.8	1.1	2.7	1.1	2.8	1.1
Tenure (year)	11.3	9.2	11.1	9.2	11.9	9.7	11.3	8.9

Note: DRP: daily rest period.

Table 2 reports the prevalence of sleep problems by DRP. The prevalence of short sleep duration, insufficient sleep, nonrestorative sleep, and not recovering from fatigue was highest among workers in workplaces with no measures, whereas the prevalence of difficulty initiating sleep, difficulty maintaining sleep, early morning awakening, and daytime sleepiness was highest among workers in workplaces with <11 h DRP.

**Table 2:** Prevalence of sleep problems by daily rest period.

	No measures		DRP<11 h		DRP≥11 h	
	N	%	N	%	N	%
Short sleep duration	2748	44.3	592	42.7	861	42.5
Insufficient sleep	2738	72.0	591	67.9	859	68.5
Difficulty initiating sleep	2748	23.0	589	23.1	861	22.4
Difficulty maintaining sleep	2746	8.5	592	11.0	861	7.5
Early morning awakening	2745	4.7	592	6.3	861	3.9
Daytime sleepiness	2746	7.6	591	8.5	860	5.1
Nonrestorative sleep	2746	22.3	588	20.9	861	19.7
Not recovering from fatigue	2737	21.0	588	19.9	855	19.3

Note: DRP: daily rest period.

Table 3 shows the logistic regression results for short sleep duration among full-time workers. In column 2, the fully adjusted model, there was no significant association between ensuring a DRP in the workplace and short sleep duration. Increasing working hours was significantly associated with higher odds of short sleep duration (OR = 1.04, 95% CI 1.03 to 1.05). Women were more likely to be short sleepers (OR = 1.26, 95% CI 1.07 to 1.47). In column 3, a likelihood ratio test suggested that the model including interaction terms had a better statistical fit than the model without interaction terms (*p* = 0.026). The interaction between DRP≥11 h and working hours was statistically significant (OR = 0.97, 95% CI 0.94 to 1.00), indicating that the association between working hours and short sleep duration appeared weaker among workers in workplaces with ≥11 h DRP. Exploratory analyses stratified by gender suggested that this pattern appeared more evident among women than among men.

**Table 3:** Daily rest periods and short sleep duration for full-time workers.

	(1)	(2)	(3)	(4)	(5)
	Full	Full	Full	Women	Men
	OR (95% CI)	OR (95% CI)	OR (95% CI)	OR (95% CI)	OR (95% CI)
DRP<11 h	0.92	0.92	0.90	1.06	0.82
	(0.76 - 1.11)	(0.76 - 1.11)	(0.74 - 1.09)	(0.81 - 1.38)	(0.61 - 1.09)
DRP≥11 h	0.94	0.93	0.93	0.93	0.91
	(0.80 - 1.11)	(0.79 - 1.10)	(0.79 - 1.10)	(0.74 - 1.17)	(0.71 - 1.16)
Weekly working hours	1.03***	1.04***	1.04***	1.04***	1.04***
	(1.02 - 1.05)	(1.03 - 1.05)	(1.03 - 1.06)	(1.02 - 1.06)	(1.02 - 1.06)
Women	1.40***	1.26**	1.26**		
	(1.23 - 1.59)	(1.07 - 1.47)	(1.07 - 1.47)		
DRP<11 h*Weekly working hours			1.01	1.05	1.00
			(0.98 - 1.04)	(1.00 - 1.10)	(0.96 - 1.04)
DRP≥11 h*Weekly working hours			0.97*	0.95*	0.98
			(0.94 - 1.00)	(0.90 - 0.99)	(0.95 - 1.02)

Observations	4201	4201	4201	2160	2041
Pseudo R-squared	0.0104	0.0232	0.0245	0.0288	0.0243

Notes: DRP: daily rest period. ***p < 0.001, **p < 0.01, *p < 0.05. Column 1: crude model. Columns 2-5: adjusted for age, education, marital status, family size, health behaviors, tenure, shift work, and occupation.

Fig. 3 illustrates the predicted probability of short sleep duration by DRP at 6-h intervals (approximately 1 SD) for women and men separately. For women in workplaces with no measures or <11 h DRP, the probability of short sleep duration increased with increasing working hours, with women in workplaces with <11 h DRP having the highest probability when weekly working hours were more than approximately 43 h. By contrast, for women in workplaces with ≥11 h DRP, the probability of short sleep duration showed a declining trend. The interaction was statistically significant (*p* = 0.005). Pairwise comparisons (Bonferroni-adjusted) suggested that the difference between <11 h DRP and ≥11 h DRP was statistically significant (*z* = 3.15, *p* = 0.005), whereas the difference between no measures and ≥11 h DRP did not reach statistical significance at the 5% level, despite showing a trend consistent with the main findings.

**Fig. 3:**
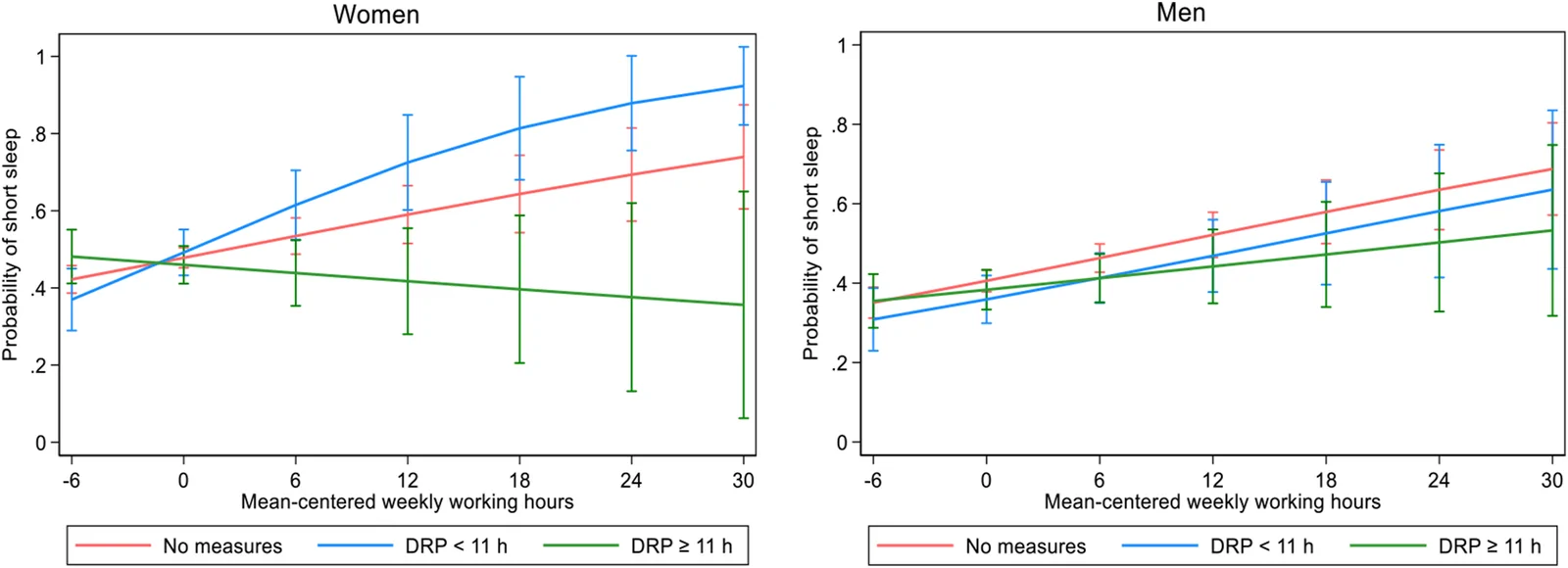
Predicted probability of short sleep duration by DRP. Notes: Predicted probabilities with 95% CIs are displayed. Weekly working hours are mean-centered, with 0 representing approximately 43 h.

For men, the probability of short sleep duration increased with increasing working hours regardless of DRPs, with those in workplaces with no measures to ensure a DRP showing the highest probability across all working hours. When weekly working hours were less than approximately 49 h, the probability was lowest among men in workplaces with <11 h DRP. However, when weekly working hours were more than approximately 49 h, the probability was lowest among men in workplaces with ≥11 h DRP. The interaction was not statistically significant (*p* = 0.529), and the probability of short sleep duration did not differ significantly between DRP groups.

We conducted several robustness checks that accounted for potential confounding factors, including workplace scale and sector, job stress, health status, household income, and time allocation for unpaid work (housework, childcare, and caregiving). As work styles may differ substantially between shift and non-shift workers, we also conducted an analysis excluding shift workers. Overall, these different specifications yielded consistent results. Details are provided in Table S1 and S2 (Supplemental Material).

### 3.1 Overtime workers

To examine whether the associations between DRPs and short sleep duration were stronger among workers particularly vulnerable to restricted sleep opportunities, we repeated the analyses as in Table 3 by focusing on overtime workers (>40 h per week). The results are presented in Table 4. In column 3, ensuring ≥11 h DRP in the workplace was significantly associated with lower odds of short sleep duration among overtime workers (OR = 0.78, 95% CI 0.61 to 0.99). An increase in overtime hours was significantly associated with higher odds of short sleep duration (OR = 1.04, 95% CI 1.02 to 1.06). Women were more likely to be short sleepers (OR = 1.33, 95% CI 1.05 to 1.68). The interactions between DRPs and overtime hours were not statistically significant. Exploratory analyses stratified by gender indicated that an increase in overtime hours was significantly associated with higher odds of short sleep duration for both women and men; however, this association appeared weaker among women in workplaces with ≥11 h DRP.

**Table 4:** Daily rest periods and short sleep duration for overtime workers.

	(1)	(2)	(3)	(4)	(5)
	Full	Full	Full	Women	Men
	OR (95% CI)	OR (95% CI)	OR (95% CI)	OR (95% CI)	OR (95% CI)
DRP<11 h	0.99	0.99	0.98	1.40	0.77
	(0.77 - 1.28)	(0.76 - 1.28)	(0.75 - 1.27)	(0.92 - 2.13)	(0.54 - 1.11)
DRP≥11 h	0.78*	0.78*	0.78*	0.74	0.77
	(0.61 - 0.99)	(0.61 - 1.00)	(0.61 - 0.99)	(0.50 - 1.08)	(0.55 - 1.08)
Overtime hours	1.03***	1.03***	1.04***	1.05*	1.03*
	(1.02 - 1.05)	(1.02 - 1.05)	(1.02 - 1.06)	(1.01 - 1.09)	(1.00 - 1.06)
Women	1.40***	1.33*	1.33*		
	(1.16 - 1.69)	(1.05 - 1.69)	(1.05 - 1.68)		
DRP<11 h*Overtime hours			1.01	1.03	1.01
			(0.97 - 1.05)	(0.95 - 1.11)	(0.97 - 1.06)
DRP≥11 h*Overtime hours			0.97	0.92*	1.00
			(0.93 - 1.01)	(0.85 - 0.99)	(0.95 - 1.04)

Observations	1927	1927	1927	826	1101
Pseudo R-squared	0.0114	0.0312	0.0326	0.0515	0.0318

Notes: DRP: daily rest period. ***p < 0.001, **p < 0.01, *p < 0.05. Column 1: crude model. Columns 2-5: adjusted for age, education, marital status, family size, health behaviors, tenure, shift work, and occupation.

### 3.2 Other sleep outcomes

Table 5 presents the results for other sleep outcomes among full-time workers. In Panel A, for the full sample, ensuring ≥11 h DRP was significantly associated with lower odds of daytime sleepiness (OR = 0.68, 95% CI 0.48 to 0.95). For the other sleep outcomes, although the associations were in the expected direction, they did not reach statistical significance. Increasing working hours was significantly associated with higher odds of insufficient sleep, daytime sleepiness, nonrestorative sleep, and feelings of not recovering from fatigue. Compared to men, women were more likely to experience sleep complaints, including insufficient sleep, difficulty initiating sleep, nonrestorative sleep, and feelings of not recovering from fatigue. The interactions between DRPs and working hours were not statistically significant for any of these outcomes.

**Table 5:** Daily rest periods and other sleep outcomes for full-time workers.

	(1)	(2)	(3)	(4)	(5)	(6)	(7)
	Insufficient sleep	Difficulty initiating sleep	Difficulty maintaining sleep	Early morning awakening	Daytime sleepiness	Nonrestorative sleep	Not recovering from fatigue
	OR (95% CI)	OR (95% CI)	OR (95% CI)	OR (95% CI)	OR (95% CI)	OR (95% CI)	OR (95% CI)
**Panel A: Full**							
DRP<11 h	0.83	1.03	1.32	1.24	1.15	0.95	0.94
	(0.68 - 1.02)	(0.82 - 1.29)	(0.96 - 1.81)	(0.82 - 1.86)	(0.82 - 1.62)	(0.75 - 1.20)	(0.74 - 1.21)
DRP≥11 h	0.87	0.97	0.83	0.78	0.68*	0.87	0.90
	(0.72 - 1.04)	(0.80 - 1.18)	(0.63 - 1.11)	(0.53 - 1.15)	(0.48 - 0.95)	(0.71 - 1.07)	(0.74 - 1.11)
Weekly working hours	1.04***	1.00	1.02	1.01	1.04***	1.05***	1.05***
	(1.02 - 1.06)	(0.98 - 1.01)	(0.99 - 1.04)	(0.98 - 1.04)	(1.02 - 1.06)	(1.03 - 1.06)	(1.03 - 1.06)
Women	1.36***	1.34**	1.10	0.76	1.29	1.99***	1.80***
	(1.15 - 1.61)	(1.11 - 1.62)	(0.83 - 1.47)	(0.52 - 1.11)	(0.93 - 1.78)	(1.63 - 2.43)	(1.48 - 2.20)
DRP<11 h*Weekly working hours	0.99	0.97	1.00	1.03	0.97	0.97	0.98
	(0.96 - 1.03)	(0.93 - 1.01)	(0.95 - 1.04)	(0.97 - 1.08)	(0.92 - 1.01)	(0.94 - 1.00)	(0.95 - 1.01)
DRP≥11 h*Weekly working hours	1.00	1.01	1.01	1.02	1.00	0.99	1.01
	(0.97 - 1.03)	(0.98 - 1.04)	(0.97 - 1.05)	(0.95 - 1.08)	(0.96 - 1.05)	(0.96 - 1.02)	(0.98 - 1.04)

Observations	4188	4198	4199	4198	4197	4195	4180
Pseudo R-squared	0.0298	0.0375	0.0323	0.0421	0.0243	0.0377	0.0271

**Panel B: Women**							
DRP<11 h	1.04	1.04	1.21	1.37	1.32	1.03	1.13
	(0.76 - 1.43)	(0.75 - 1.44)	(0.78 - 1.89)	(0.72 - 2.61)	(0.82 - 2.13)	(0.75 - 1.41)	(0.82 - 1.55)
DRP≥11 h	0.97	1.06	0.90	0.86	0.75	0.75*	0.91
	(0.73 - 1.28)	(0.81 - 1.39)	(0.61 - 1.33)	(0.46 - 1.61)	(0.48 - 1.17)	(0.57 - 0.99)	(0.70 - 1.20)
Weekly working hours	1.03	0.99	1.01	0.96	1.04*	1.03**	1.05***
	(1.00 - 1.06)	(0.96 - 1.01)	(0.97 - 1.06)	(0.91 - 1.02)	(1.01 - 1.07)	(1.01 - 1.06)	(1.02 - 1.07)
DRP<11 h*Weekly working hours	0.99	1.00	1.00	1.13**	0.97	1.00	1.01
	(0.94 - 1.04)	(0.95 - 1.05)	(0.93 - 1.07)	(1.04 - 1.24)	(0.91 - 1.03)	(0.95 - 1.06)	(0.96 - 1.06)
DRP≥11 h*Weekly working hours	1.03	1.01	1.01	0.98	0.98	0.99	1.00
	(0.96 - 1.09)	(0.96 - 1.06)	(0.93 - 1.09)	(0.85 - 1.12)	(0.92 - 1.05)	(0.95 - 1.04)	(0.95 - 1.05)

Observations	2153	2159	2160	2159	2159	2157	2149
Pseudo R-squared	0.0239	0.0357	0.0424	0.0749	0.0383	0.0348	0.0266

**Panel C: Men**							
DRP<11 h	0.67**	1.06	1.36	1.25	0.85	0.93	0.76
	(0.51 - 0.89)	(0.78 - 1.46)	(0.85 - 2.17)	(0.75 - 2.09)	(0.51 - 1.43)	(0.63 - 1.38)	(0.52 - 1.10)
DRP≥11 h	0.82	0.87	0.78	0.65	0.56*	1.11	0.89
	(0.64 - 1.04)	(0.65 - 1.17)	(0.50 - 1.21)	(0.36 - 1.17)	(0.33 - 0.97)	(0.81 - 1.53)	(0.65 - 1.22)
Weekly working hours	1.04***	1.00	1.02	1.02	1.04*	1.06***	1.04***
	(1.02 - 1.07)	(0.98 - 1.02)	(0.99 - 1.05)	(0.99 - 1.05)	(1.01 - 1.07)	(1.04 - 1.08)	(1.02 - 1.07)
DRP<11 h*Weekly working hours	1.01	0.95	1.00	0.98	0.97	0.95*	0.97
	(0.97 - 1.05)	(0.90 - 1.00)	(0.94 - 1.06)	(0.91 - 1.05)	(0.91 - 1.04)	(0.91 - 1.00)	(0.93 - 1.02)
DRP≥11 h*Weekly working hours	0.99	1.01	1.01	1.03	1.03	0.98	1.01
	(0.96 - 1.03)	(0.98 - 1.05)	(0.96 - 1.06)	(0.97 - 1.09)	(0.97 - 1.08)	(0.94 - 1.02)	(0.98 - 1.06)

Observations	2035	2039	2039	2039	2011	2038	2031
Pseudo R-squared	0.0330	0.0433	0.0442	0.0554	0.0368	0.0374	0.0285

Notes: DRP: daily rest period. ***p < 0.001, **p < 0.01, *p < 0.05. All models adjusted for age, education, marital status, family size, health behaviors, tenure, shift work, and occupation.

Subgroup analyses stratified by gender suggested gender-specific differences. In Panel B, for women, ensuring ≥11 h DRP was significantly associated with lower odds of nonrestorative sleep (OR = 0.75, 95% CI 0.57 to 0.99). Increasing working hours was significantly associated with higher odds of daytime sleepiness, nonrestorative sleep, and feelings of not recovering from fatigue. Moreover, the association between working hours and early morning awakening appeared stronger among women in workplaces with <11 h DRP. In Panel C, for men, ensuring <11 h DRP was significantly associated with lower odds of insufficient sleep (OR = 0.67, 95% CI 0.51 to 0.89), whereas ensuring ≥11 h DRP was significantly associated with lower odds of daytime sleepiness (OR = 0.56, 95% CI 0.33 to 0.97). Increasing working hours was significantly associated with higher odds of insufficient sleep, daytime sleepiness, nonrestorative sleep, and feelings of not recovering from fatigue. Moreover, the association between working hours and nonrestorative sleep appeared weaker among men in workplaces with <11 h DRP.

## 4 Discussion

To the best of our knowledge, this study is the first to utilize matched employer**–**employee survey data to examine the association between ensuring a DRP in the workplace and workers’ sleep. We found no significant association between ensuring a DRP in the workplace and short sleep duration among full-time workers. Increasing working hours was significantly associated with higher odds of short sleep duration; however, this association appeared weaker among workers in workplaces with ≥11 h DRP. Exploratory analyses stratified by gender suggested that this pattern appeared more evident among women than among men. Moreover, ensuring ≥11 h DRP in the workplace was significantly associated with lower odds of short sleep duration among overtime workers. These findings are broadly consistent with previous research. Specifically, sleep duration has been reported to be significantly associated with DRP variation (Kubo et al., 2018), and a longer DRP has been found to be associated with increased time spent in bed or longer sleep duration (Ikeda et al., 2018, 2022; Kubo et al., 2018). Similarly, an intra-individual field study utilizing objective sleep data found that quick returns were associated with 1 h shorter sleep length among Swedish nurses (Öster et al., 2024).

This study evaluated workplace-level DRPs based on information collected in the employer survey, rather than on the actual DRPs self-reported by individual workers. Workers' actual DRPs may vary daily, depending on workload. By contrast, ensuring a DRP in the workplace secures a minimum rest duration for workers, which may play a vital role, especially when workloads are heavy. Therefore, our findings should be interpreted as associations between workplace-guaranteed minimum DRPs and workers’ sleep outcomes, rather than the actual DRPs experienced by workers, which have been the predominant focus of prior research.

In addition, ensuring ≥11 h DRP in the workplace was significantly associated with lower odds of daytime sleepiness; however, the findings for other sleep outcomes were not statistically significant, although they were in the hypothesized direction. Prior studies using a sleep quality index generally reported that longer DRPs were associated with improved sleep quality. For instance, among Japanese daytime workers, shorter DRPs were associated with poorer sleep quality, as measured by the Pittsburgh Sleep Quality Index (PSQI) (Ikeda et al., 2018). A longitudinal study demonstrated that extending the DRP by 2 h or longer was associated with improved PSQI scores (Ikeda et al., 2022). In an intra-individual field study of Swedish nurses, quick returns were associated with reduced subjective sleep quality (Öster et al., 2024).

Moreover, the findings regarding daytime sleepiness align with previous cross-sectional studies in the healthcare sector. Specifically, quick returns were associated with excessive sleepiness among Norwegian nurses (Eldevik et al., 2013) and with increased worktime sleepiness among Swedish nurses (Öster et al., 2024). However, a longitudinal study suggested no association between quick returns and excessive sleepiness among Norwegian nurses (Flo et al., 2014).

For fatigue-related outcomes, our findings diverge from the existing literature. A one-month observational study using a fatigue app among Japanese information technology workers reported that longer DRPs were associated with improved fatigue recovery and psychological detachment from work (Kubo et al., 2018). Similarly, a longitudinal study of Norwegian nurses demonstrated that fewer quick returns were related to reduced risk of pathological fatigue (Flo et al., 2014). By contrast, this study found no statistically significant association between workplace-level DRPs and nonrestorative sleep or feelings of not recovering from fatigue in the full sample.

The finding that increasing working hours was significantly associated with higher odds of various sleep problems is consistent with previous research conducted in Japan, which measured working hours using objective attendance records (Nakashima et al., 2011). While this prior study could not account for job stress due to data constraints, the present study addressed this limitation. Specifically, we confirmed that a significant association persisted between increasing working hours and short sleep duration, even after accounting for job stress in terms of quantitative overload (Table S2). Moreover, increasing working hours remained significantly associated with higher odds of insufficient sleep, daytime sleepiness, nonrestorative sleep, and feelings of not recovering from fatigue, even after controlling for quantitative overload (Table S5).

Previous research has documented that women report more sleep complaints than men (Arber et al., 2009; Groeger et al., 2004; Sekine et al., 2006). In Japan, compared to men, women reported shorter sleep duration (Li et al., 2021; Robinson & Michelson, 2010; Tsunoda et al., 2015). By contrast, although men and women in the UK exhibited similar sleep duration, women still reported a higher prevalence of sleep problems (Groeger et al., 2004). Consistent with prior evidence, our findings demonstrated pronounced gender differences, with women more likely to experience short sleep duration, insufficient sleep, difficulty initiating sleep, nonrestorative sleep, and feelings of not recovering from fatigue. Meanwhile, among women in workplaces with <11 h DRP, the association between increasing working hours and early morning awakening appeared stronger. Conversely, among women in workplaces with ≥11 h DRP, the association between increasing working hours and short sleep duration appeared weaker and the odds of nonrestorative sleep were lower.

Fig. 3 illustrates an increasing trend in the probability of short sleep duration with increasing working hours for women in workplaces with <11 h DRP, in contrast to a declining trend for those in workplaces with ≥11 h DRP. One possible explanation is that women with short DRPs may have to shorten sleep to perform domestic duties, whereas women with sufficient DRPs may not need to do so. In addition, long working hours cause fatigue, which generates a greater need for fatigue recovery (Vieten et al., 2022). Despite this critical need, restricted sleep opportunities combined with a high likelihood of experiencing sleep problems, such as early morning awakening, may result in short sleep duration. Conversely, given sufficient sleep opportunities and the absence of such sleep disruptions, women may achieve longer sleep duration.

While men in workplaces with <11 h DRP exhibited lower odds of insufficient sleep and a weaker association between increasing working hours and nonrestorative sleep, those in workplaces with ≥11 h DRP showed lower odds of daytime sleepiness.

Overall, the findings support the practical relevance of the 11 h DRP framework as a policy-based benchmark; however, 11 h should not be interpreted as a definitive threshold. Analyses using an alternative cutoff of 12 h demonstrated consistent but more pronounced associations with short sleep duration (Table S3) and nonrestorative sleep among women (Table S4), indicating that longer DRPs may yield stronger associations with favorable sleep outcomes, particularly for women. Therefore, future research should further investigate whether ensuring a minimum of 11 h DRP is sufficient or optimal.

This study has several limitations. First, due to its cross-sectional design, this study could not identify causal relationships. Second, unobserved workplace characteristics such as the workplace's philosophy regarding work-life balance, labor shortages, and business volume, may influence both the implementation of measures to ensure a DRP and workers' working hours, potentially biasing the results. Third, workplace-guaranteed DRPs may not reflect the actual DRPs available for workers. For instance, long commuting times or work-related calls and emails during off-hours could encroach upon the time available for rest. Fourth, although the employee questionnaires were randomly distributed, a relatively high percentage of women and clerical workers participated in the survey, indicating potential sampling bias. Fifth, this study relied on self-reported working hours and sleep measures. Future studies using workplace attendance records and objective sleep measures may provide more precise insights.

## 5 Conclusions

Using cross-sectional data from a matched employer-employee survey, we found that increasing working hours was significantly associated with higher odds of short sleep duration; however, this association appeared weaker among workers in workplaces with ≥11 h DRP, particularly among women. Moreover, ensuring ≥11 h DRP was significantly associated with lower odds of short sleep duration among overtime workers and daytime sleepiness among full-time workers. Gender-specific differences were observed in the associations between ensuring a DRP and other sleep problems, such as insufficient sleep, early morning awakening, daytime sleepiness, and nonrestorative sleep. Overall, these findings support the relevance of introducing a work-interval system or taking measures to ensure a DRP in the workplace for workers’ sleep health. Nevertheless, further longitudinal, interventional, or quasi-experimental studies are required to establish the causal relationship between ensuring a DRP in the workplace and subsequent sleep outcomes. Furthermore, although ensuring ≥11 h DRP serves as a practical, policy-based benchmark, future studies should explore whether 11 h is a sufficient or optimal threshold.

## CRediT authorship contribution statement

**Huilin Wang:** Writing – review & editing, Writing – original draft, Software, Methodology, Formal analysis, Data curation, Conceptualization. **Hideyuki Kobayashi:** Writing – review & editing, Project administration, Investigation, Data curation. **Masaya Takahashi:** Writing – review & editing, Supervision, Conceptualization.

## Ethics approval and consent to participate

Ethical approval was obtained from the Research Ethics Committee of the National Institute of Occupational Safety and Health, Japan (No. 2022N23). Respondents were informed that participation in the survey was voluntary, and that the data would remain anonymous, impersonal, and would be used only for scientific purposes.

## Funding

Not applicable.

## Conflict of interest

None to declare.

## Data Availability

Data will be made available upon reasonable request.
