# Supplementary material for: Association between ensuring a daily rest period in the workplace and workers’ sleep: Evidence from a matched employer–employee survey in Japan
Source: SSM Popul Health. 2026 Sep 14;36:101967. doi: 10.1016/j.ssmph.2026.101967 (PMC13657113; doi:10.1016/j.ssmph.2026.101967)
Supplement: Supplementary file 1 [file mmc1.docx]

**Supplemental Material**

**Association between ensuring a daily rest period in the workplace and workers’ sleep: Evidence from a matched employer–employee survey in Japan**

**S1. Robustness checks**

As robustness checks, we accounted for the following potential confounding factors. First, the prevalence of demanding work schedules may vary greatly across sectors. Sectors such as healthcare, transportation, and services are characterized by long working hours and nonstandard work schedules. In column 1, we additionally controlled for workplace sector and scale.

Second, job stress may be correlated with working hours and sleep duration; therefore, in column 2, the analysis controlled for job stress using a subscale of the Brief Job Stress Questionnaire that assesses quantitative overload. This subscale consists of three items: “I have an extremely large amount of work to do,” “I can't complete work in the required time,” and “I have to work as hard as I can.” The response options were as follows: very much so, moderately so, somewhat, and not at all. The options were reverse-coded from 1 to 4, and the three items were then summed to produce a total score ranging from 3 to 12, with higher scores indicating greater quantitative overload.

Third, health status and household income may influence both working hours and sleep duration; therefore, these factors were controlled for in column 3. For health status, respondents were asked whether in the past year, they had been diagnosed with or treated for any of the 17 listed diseases or any other unlisted conditions. The listed diseases included hypertension, diabetes, hyperlipidemia, obesity, hyperuricemia, stroke, heart disease, arrhythmia, liver disease, kidney disease, duodenal ulcer, gastric ulcer, cancer, asthma, insomnia, metabolic syndrome, and depression. The total number of diseases was used as an indicator of health status.

Fourth, intra-household duties may influence individuals’ market working hours and sleep duration; therefore, unpaid work hours (housework, childcare, and caregiving) were controlled for in column 4. Respondents were asked, “In the past month, on average, how many hours did you spend on housework, childcare, and caregiving on workdays, respectively?” The response options were: 0 h, <1 h, 1–2 h, 2–3 h, 3–4 h, 4–5 h, and ≥5 h, coded as 0 to 6, respectively. Housework, childcare, and caregiving were treated as continuous variables in the analysis.

Finally, given that work styles may differ substantially between shift and non-shift workers, shift workers were excluded from the analysis in column 5.

Descriptive statistics for the variables used in the robustness checks and the estimation results are provided in Tables S1 and S2.

| Table S1 Descriptive Statistics for the variables used in the robustness checks. | | | | | | | | | | | | | | | |
| --- | --- | --- | --- | --- | --- | --- | --- | --- | --- | --- | --- | --- | --- | --- | --- |
|  | **Full sample** | | |  | **No measures** | | |  | **DRP<11 h** | | |  | **DRP≥11 h** | | |
|  | N | n | % |  | N | n | % |  | N | n | % |  | N | n | % |
| **Sector** | 4,202 |  |  |  | 2,749 |  |  |  | 592 |  |  |  | 861 |  |  |
| Manufacturing |  | 861 | 20.5 |  |  | 546 | 19.9 |  |  | 123 | 20.8 |  |  | 192 | 22.3 |
| Construction |  | 493 | 11.7 |  |  | 341 | 12.4 |  |  | 77 | 13.0 |  |  | 75 | 8.7 |
| Information media and telecommunications |  | 103 | 2.5 |  |  | 62 | 2.3 |  |  | 29 | 4.9 |  |  | 12 | 1.4 |
| Transportation and postal services |  | 140 | 3.3 |  |  | 51 | 1.9 |  |  | 57 | 9.6 |  |  | 32 | 3.7 |
| Wholesale and retail trade |  | 565 | 13.4 |  |  | 378 | 13.8 |  |  | 59 | 10.0 |  |  | 128 | 14.9 |
| Finance, insurance, real estate, rental and leasing |  | 116 | 2.8 |  |  | 82 | 3.0 |  |  | 10 | 1.7 |  |  | 24 | 2.8 |
| Scientific research, professional/technical services |  | 107 | 2.5 |  |  | 71 | 2.6 |  |  | 19 | 3.2 |  |  | 17 | 2.0 |
| Accommodation, living-related/entertainment services | | 508 | 12.1 |  |  | 337 | 12.3 |  |  | 88 | 14.9 |  |  | 83 | 9.6 |
| Education, learning support |  | 221 | 5.3 |  |  | 169 | 6.1 |  |  | 12 | 2.0 |  |  | 40 | 4.6 |
| Healthcare and social assistance |  | 895 | 21.3 |  |  | 584 | 21.2 |  |  | 89 | 15.0 |  |  | 222 | 25.8 |
| Other |  | 193 | 4.6 |  |  | 128 | 4.7 |  |  | 29 | 4.9 |  |  | 36 | 4.2 |
| **Workplace scale** | 4,202 |  |  |  | 2,749 |  |  |  | 592 |  |  |  | 861 |  |  |
| Less than 10 |  | 303 | 7.2 |  |  | 217 | 7.9 |  |  | 35 | 5.9 |  |  | 51 | 5.9 |
| 10~29 |  | 1,833 | 43.6 |  |  | 1,205 | 43.8 |  |  | 251 | 42.4 |  |  | 377 | 43.8 |
| 30~49 |  | 856 | 20.4 |  |  | 575 | 20.9 |  |  | 98 | 16.6 |  |  | 183 | 21.3 |
| 50~99 |  | 657 | 15.6 |  |  | 412 | 15.0 |  |  | 103 | 17.4 |  |  | 142 | 16.5 |
| 100~299 |  | 419 | 10.0 |  |  | 253 | 9.2 |  |  | 79 | 13.3 |  |  | 87 | 10.1 |
| 300 or more |  | 134 | 3.2 |  |  | 87 | 3.2 |  |  | 26 | 4.4 |  |  | 21 | 2.4 |
| **Household income (Ten thousand yen)** | 4,191 |  |  |  | 2,742 |  |  |  | 591 |  |  |  | 858 |  |  |
| Less than 400 |  | 834 | 19.9 |  |  | 546 | 19.9 |  |  | 113 | 19.1 |  |  | 175 | 20.4 |
| 400~599 |  | 1,112 | 26.5 |  |  | 728 | 26.5 |  |  | 160 | 27.1 |  |  | 224 | 26.1 |
| 600~799 |  | 737 | 17.6 |  |  | 479 | 17.5 |  |  | 102 | 17.3 |  |  | 156 | 18.2 |
| 800 or higher |  | 761 | 18.2 |  |  | 501 | 18.3 |  |  | 115 | 19.5 |  |  | 145 | 16.9 |
| Prefer not to answer |  | 747 | 17.8 |  |  | 488 | 17.8 |  |  | 101 | 17.1 |  |  | 158 | 18.4 |
|  | N | Mean | SD |  | N | Mean | SD |  | N | Mean | SD |  | N | Mean | SD |
| Health status | 4,202 | 0.7 | 0.9 |  | 2,749 | 0.6 | 0.9 |  | 592 | 0.6 | 0.9 |  | 861 | 0.7 | 1.0 |
| Quantitative load | 4,142 | 8.0 | 2.0 |  | 2,708 | 8.0 | 2.1 |  | 584 | 7.9 | 2.0 |  | 850 | 7.9 | 2.0 |
| Housework | 4,185 | 2.6 | 1.8 |  | 2,735 | 2.6 | 1.8 |  | 591 | 2.5 | 1.8 |  | 859 | 2.6 | 1.8 |
| Childcare | 4,106 | 0.9 | 1.7 |  | 2,676 | 0.9 | 1.7 |  | 586 | 0.8 | 1.6 |  | 844 | 0.9 | 1.7 |
| Caregiving | 4,067 | 0.2 | 0.8 |  | 2,647 | 0.2 | 0.9 |  | 580 | 0.2 | 0.8 |  | 840 | 0.1 | 0.7 |
| Note: DRP: daily rest period. |  |  |  |  |  |  |  |  |  |  |  |  |  |  |  |

| Table S2 Daily rest periods and short sleep duration for full-time workers－robustness checks. | | | |  |  |
| --- | --- | --- | --- | --- | --- |
|  | Sector and scale | Quantitative overload | Health status and household income | Unpaid work | Excluding shift workers |
|  | OR  (95% CI) | OR  (95% CI) | OR  (95% CI) | OR  (95% CI) | OR  (95% CI) |
| **Panel A: Full** |  |  |  |  |  |
| DRP<11 h | 0.88 | 0.92 | 0.91 | 0.91 | 0.89 |
|  | (0.73 - 1.07) | (0.76 - 1.12) | (0.75 - 1.10) | (0.75 - 1.10) | (0.74 - 1.08) |
| DRP≥11 h | 0.93 | 0.93 | 0.93 | 0.96 | 0.90 |
|  | (0.78 - 1.09) | (0.79 - 1.10) | (0.79 - 1.09) | (0.81 - 1.14) | (0.76 - 1.07) |
| Weekly working hours | 1.04*** | 1.03*** | 1.04*** | 1.04*** | 1.04*** |
|  | (1.03 - 1.06) | (1.02 - 1.05) | (1.03 - 1.06) | (1.03 - 1.06) | (1.03 - 1.06) |
| Women | 1.28** | 1.26** | 1.26** | 1.17 | 1.24** |
|  | (1.09 - 1.51) | (1.07 - 1.48) | (1.07 - 1.49) | (0.99 - 1.38) | (1.06 - 1.46) |
| DRP<11 h*Weekly working hours | 1.01 | 1.01 | 1.01 | 1.01 | 1.01 |
|  | (0.98 - 1.04) | (0.98 - 1.04) | (0.98 - 1.04) | (0.98 - 1.04) | (0.98 - 1.04) |
| DRP≥11 h*Weekly working hours | 0.97* | 0.97* | 0.97* | 0.97* | 0.97* |
|  | (0.94 - 1.00) | (0.94 - 1.00) | (0.94 - 1.00) | (0.94 - 1.00) | (0.94 - 0.99) |
| Observations | 4,201 | 4,141 | 4,190 | 4,047 | 4,101 |
| Pseudo R-squared | 0.0271 | 0.0301 | 0.0283 | 0.0255 | 0.0252 |
| **Panel B: Women** |  |  |  |  |  |
| DRP<11 h | 1.00 | 1.07 | 1.07 | 1.08 | 1.03 |
|  | (0.76 - 1.32) | (0.82 - 1.40) | (0.82 - 1.39) | (0.82 - 1.42) | (0.79 - 1.35) |
| DRP≥11 h | 0.91 | 0.92 | 0.91 | 0.97 | 0.87 |
|  | (0.72 - 1.14) | (0.73 - 1.16) | (0.72 - 1.14) | (0.76 - 1.22) | (0.69 - 1.10) |
| Weekly working hours | 1.04*** | 1.03** | 1.04*** | 1.04*** | 1.04*** |
|  | (1.02 - 1.07) | (1.01 - 1.06) | (1.02 - 1.06) | (1.02 - 1.07) | (1.02 - 1.07) |
| DRP<11 h*Weekly working hours | 1.04 | 1.04 | 1.05 | 1.04 | 1.04 |
|  | (0.99 - 1.10) | (0.99 - 1.10) | (0.99 - 1.10) | (0.99 - 1.10) | (0.99 - 1.10) |
| DRP≥11 h*Weekly working hours | 0.95* | 0.95* | 0.95* | 0.95* | 0.95* |
|  | (0.90 - 0.99) | (0.91 - 1.00) | (0.91 - 1.00) | (0.91 - 1.00) | (0.90 - 0.99) |
| Observations | 2,160 | 2,134 | 2,153 | 2,070 | 2,087 |
| Pseudo R-squared | 0.0338 | 0.0334 | 0.0332 | 0.0341 | 0.0289 |
| **Panel C: Men** |  |  |  |  |  |
| DRP<11 h | 0.81 | 0.83 | 0.82 | 0.81 | 0.82 |
|  | (0.60 - 1.08) | (0.62 - 1.12) | (0.61 - 1.09) | (0.61 - 1.08) | (0.62 - 1.10) |
| DRP≥11 h | 0.92 | 0.92 | 0.92 | 0.93 | 0.92 |
|  | (0.72 - 1.17) | (0.72 - 1.18) | (0.72 - 1.18) | (0.72 - 1.19) | (0.72 - 1.17) |
| Weekly working hours | 1.04*** | 1.03** | 1.04*** | 1.04*** | 1.04*** |
|  | (1.02 - 1.06) | (1.01 - 1.05) | (1.02 - 1.06) | (1.02 - 1.06) | (1.02 - 1.06) |
| DRP<11 h*Weekly working hours | 1.01 | 1.00 | 1.00 | 1.00 | 1.00 |
|  | (0.97 - 1.04) | (0.97 - 1.04) | (0.96 - 1.04) | (0.96 - 1.04) | (0.96 - 1.03) |
| DRP≥11 h*Weekly working hours | 0.98 | 0.98 | 0.98 | 0.98 | 0.98 |
|  | (0.95 - 1.02) | (0.95 - 1.02) | (0.95 - 1.02) | (0.95 - 1.02) | (0.94 - 1.01) |
| Observations | 2,041 | 2,007 | 2,037 | 1,977 | 2,014 |
| Pseudo R-squared | 0.0312 | 0.0314 | 0.0301 | 0.0235 | 0.0249 |
| Notes: DRP: daily rest period. *** p<0.001, ** p<0.01, * p<0.05. All models adjusted for age, education, marital status, family size, health behaviors, tenure, shift work, and occupation. | | | | | |

**S2. Alternative cutoff**

| Table S3 Daily rest periods and short sleep durationー12 h cutoff. | | |  |  |  |
| --- | --- | --- | --- | --- | --- |
|  | Full | Full | Full | Women | Men |
|  | OR  (95% CI) | OR  (95% CI) | OR  (95% CI) | OR  (95% CI) | OR  (95% CI) |
| **Panel A: Full-time workers** |  |  |  |  |  |
| DRP<12 h | 0.95 | 0.95 | 0.95 | 1.05 | 0.89 |
|  | (0.81 - 1.11) | (0.81 - 1.11) | (0.81 - 1.11) | (0.84 - 1.31) | (0.69 - 1.13) |
| DRP≥12 h | 0.90 | 0.89 | 0.88 | 0.84 | 0.85 |
|  | (0.74 - 1.10) | (0.73 - 1.09) | (0.72 - 1.08) | (0.63 - 1.13) | (0.63 - 1.14) |
| Weekly working hours | 1.03*** | 1.04*** | 1.04*** | 1.04*** | 1.04*** |
|  | (1.02 - 1.05) | (1.03 - 1.05) | (1.03 - 1.06) | (1.02 - 1.06) | (1.02 - 1.06) |
| Women | 1.40*** | 1.26** | 1.26** |  |  |
|  | (1.23 - 1.59) | (1.07 - 1.47) | (1.07 - 1.48) |  |  |
| DRP<12 h*Weekly working hours |  |  | 1.00 | 1.03 | 0.99 |
|  |  |  | (0.98 - 1.03) | (0.98 - 1.07) | (0.96 - 1.02) |
| DRP≥12 h*Weekly working hours |  |  | 0.96* | 0.91** | 0.98 |
|  |  |  | (0.93 - 1.00) | (0.86 - 0.97) | (0.94 - 1.02) |
| Observations | 4,201 | 4,201 | 4,201 | 2,160 | 2,041 |
| Pseudo R-squared | 0.0104 | 0.0232 | 0.0243 | 0.0291 | 0.0240 |
| **Panel B: Overtime workers** |  |  |  |  |  |
| DRP<12 h | 0.96 | 0.96 | 0.96 | 1.28 | 0.79 |
|  | (0.78 - 1.20) | (0.77 - 1.20) | (0.77 - 1.20) | (0.91 - 1.79) | (0.58 - 1.08) |
| DRP≥12 h | 0.70* | 0.70* | 0.69* | 0.52* | 0.73 |
|  | (0.51 - 0.95) | (0.51 - 0.95) | (0.51 - 0.94) | (0.31 - 0.87) | (0.48 - 1.10) |
| Overtime hours | 1.03*** | 1.03*** | 1.04*** | 1.05* | 1.03* |
|  | (1.02 - 1.05) | (1.02 - 1.05) | (1.02 - 1.06) | (1.01 - 1.09) | (1.00 - 1.06) |
| Women | 1.40*** | 1.33* | 1.33* |  |  |
|  | (1.16 - 1.69) | (1.05 - 1.69) | (1.05 - 1.68) |  |  |
| DRP<12 h*Overtime |  |  | 1.00 | 1.00 | 1.01 |
|  |  |  | (0.96 - 1.03) | (0.94 - 1.06) | (0.97 - 1.05) |
| DRP≥12 h*Overtime |  |  | 0.97 | 0.87* | 1.00 |
|  |  |  | (0.92 - 1.01) | (0.79 - 0.97) | (0.95 - 1.05) |
| Observations | 1,927 | 1,927 | 1,927 | 826 | 1,101 |
| Pseudo R-squared | 0.0119 | 0.0318 | 0.0326 | 0.0528 | 0.0318 |
| Notes: *** p<0.001, ** p<0.01, * p<0.05. DRP: daily rest period. Model 1: crude model. Models 2-5: adjusted for age, education, marital status, family size, health behaviors, tenure, shift work, and occupation. | | | | | |

| Table S4 Daily rest periods and other sleep outcomes for full-time workersー12 h cutoff. | | | |  |  |  |  |
| --- | --- | --- | --- | --- | --- | --- | --- |
|  | Insufficient sleep | Difficulty initiating sleep | Difficulty maintaining sleep | Early morning awakening | Daytime sleepiness | Nonrestorative sleep | Not recovering from fatigue |
|  | OR  (95% CI) | OR  (95% CI) | OR  (95% CI) | OR  (95% CI) | OR  (95% CI) | OR  (95% CI) | OR  (95% CI) |
| **Panel A: Full** |  |  |  |  |  |  |  |
| DRP<12 h | 0.85 | 1.01 | 1.12 | 0.99 | 0.93 | 0.95 | 0.97 |
|  | (0.71 - 1.02) | (0.84 - 1.22) | (0.85 - 1.47) | (0.68 - 1.44) | (0.68 - 1.26) | (0.78 - 1.16) | (0.79 - 1.19) |
| DRP≥12 h | 0.85 | 0.96 | 0.84 | 0.91 | 0.77 | 0.81 | 0.83 |
|  | (0.69 - 1.04) | (0.76 - 1.22) | (0.60 - 1.17) | (0.59 - 1.41) | (0.52 - 1.15) | (0.63 - 1.05) | (0.65 - 1.06) |
| Weekly working hours | 1.04*** | 1.00 | 1.02 | 1.01 | 1.04*** | 1.05*** | 1.05*** |
|  | (1.02 - 1.06) | (0.98 - 1.01) | (0.99 - 1.04) | (0.98 - 1.04) | (1.02 - 1.06) | (1.03 - 1.06) | (1.03 - 1.06) |
| Women | 1.36*** | 1.34** | 1.10 | 0.76 | 1.28 | 1.98*** | 1.80*** |
|  | (1.15 - 1.62) | (1.11 - 1.61) | (0.82 - 1.46) | (0.52 - 1.11) | (0.93 - 1.77) | (1.62 - 2.42) | (1.48 - 2.19) |
| DRP<12 h*Weekly working hours | 1.00 | 0.98 | 1.01 | 1.03 | 0.98 | 0.98 | 0.99 |
|  | (0.97 - 1.03) | (0.95 - 1.01) | (0.97 - 1.05) | (0.97 - 1.08) | (0.94 - 1.02) | (0.95 - 1.00) | (0.97 - 1.02) |
| DRP≥12 h*Weekly working hours | 0.99 | 1.01 | 0.98 | 1.02 | 0.99 | 0.99 | 1.00 |
|  | (0.95 - 1.02) | (0.97 - 1.04) | (0.94 - 1.03) | (0.95 - 1.09) | (0.95 - 1.04) | (0.95 - 1.03) | (0.96 - 1.03) |
| Observations | 4,188 | 4,198 | 4,199 | 4,198 | 4,197 | 4,195 | 4,180 |
| Pseudo R-squared | 0.0299 | 0.0369 | 0.0313 | 0.0400 | 0.0215 | 0.0378 | 0.0270 |
| **Panel B: Women** |  |  |  |  |  |  |  |
| DRP<12 h | 1.03 | 1.01 | 1.03 | 1.15 | 1.06 | 0.94 | 1.09 |
|  | (0.78 - 1.36) | (0.77 - 1.32) | (0.71 - 1.51) | (0.67 - 1.98) | (0.70 - 1.60) | (0.72 - 1.22) | (0.84 - 1.42) |
| DRP≥12 h | 0.90 | 1.15 | 0.94 | 1.03 | 0.81 | 0.71* | 0.82 |
|  | (0.65 - 1.23) | (0.83 - 1.58) | (0.58 - 1.53) | (0.50 - 2.12) | (0.47 - 1.38) | (0.51 - 0.98) | (0.59 - 1.15) |
| Weekly working hours | 1.03 | 0.99 | 1.01 | 0.96 | 1.04** | 1.03** | 1.05*** |
|  | (1.00 - 1.06) | (0.96 - 1.01) | (0.97 - 1.06) | (0.91 - 1.02) | (1.01 - 1.08) | (1.01 - 1.06) | (1.02 - 1.07) |
| DRP<12 h*Weekly working hours | 1.02 | 0.99 | 1.02 | 1.12** | 0.98 | 1.01 | 1.01 |
|  | (0.96 - 1.07) | (0.95 - 1.04) | (0.96 - 1.08) | (1.03 - 1.22) | (0.93 - 1.04) | (0.96 - 1.06) | (0.97 - 1.06) |
| DRP≥12 h*Weekly working hours | 0.98 | 1.03 | 0.96 | 0.99 | 0.95 | 0.97 | 0.98 |
|  | (0.92 - 1.05) | (0.97 - 1.10) | (0.87 - 1.07) | (0.85 - 1.16) | (0.88 - 1.03) | (0.91 - 1.03) | (0.91 - 1.04) |
| Observations | 2,153 | 2,159 | 2,160 | 2,159 | 2,159 | 2,157 | 2,149 |
| Pseudo R-squared | 0.0237 | 0.0362 | 0.0420 | 0.0711 | 0.0355 | 0.0348 | 0.0272 |
| **Panel C: Men** |  |  |  |  |  |  |  |
| DRP<12 h | 0.72** | 1.04 | 1.20 | 0.96 | 0.72 | 1.08 | 0.86 |
|  | (0.57 - 0.92) | (0.79 - 1.36) | (0.80 - 1.82) | (0.59 - 1.56) | (0.45 - 1.15) | (0.79 - 1.48) | (0.63 - 1.17) |
| DRP≥12 h | 0.80 | 0.80 | 0.69 | 0.79 | 0.64 | 0.97 | 0.78 |
|  | (0.60 - 1.06) | (0.55 - 1.17) | (0.41 - 1.17) | (0.41 - 1.51) | (0.34 - 1.20) | (0.65 - 1.46) | (0.53 - 1.16) |
| Weekly working hours | 1.04*** | 1.00 | 1.02 | 1.02 | 1.04* | 1.06*** | 1.04*** |
|  | (1.02 - 1.07) | (0.98 - 1.02) | (0.99 - 1.05) | (0.99 - 1.05) | (1.01 - 1.07) | (1.04 - 1.08) | (1.02 - 1.07) |
| DRP<12 h*Weekly working hours | 1.01 | 0.97 | 1.01 | 0.99 | 0.99 | 0.95** | 0.98 |
|  | (0.97 - 1.05) | (0.93 - 1.01) | (0.96 - 1.06) | (0.93 - 1.06) | (0.93 - 1.04) | (0.92 - 0.99) | (0.95 - 1.02) |
| DRP≥12 h*Weekly working hours | 0.99 | 1.00 | 1.00 | 1.02 | 1.02 | 0.99 | 1.01 |
|  | (0.95 - 1.03) | (0.96 - 1.05) | (0.94 - 1.05) | (0.96 - 1.09) | (0.96 - 1.08) | (0.95 - 1.04) | (0.97 - 1.06) |
| Observations | 2,035 | 2,039 | 2,039 | 2,039 | 2,011 | 2,038 | 2,031 |
| Pseudo R-squared | 0.0328 | 0.0416 | 0.0441 | 0.0516 | 0.0348 | 0.0373 | 0.0266 |
| Notes: *** p<0.001, ** p<0.01, * p<0.05. DRP: daily rest period. All models adjusted for age, education, marital status, family size, health behaviors, tenure, shift work, and occupation. | | | | | | | |

**S3. Job stress**

| Table S5 Daily rest periods and other sleep outcomes among full-time workers, controlling for quantitative overload. | | | | | | | |
| --- | --- | --- | --- | --- | --- | --- | --- |
|  | Insufficient sleep | Difficulty initiating sleep | Difficulty maintaining sleep | Early morning awakening | Daytime sleepiness | Nonrestorative sleep | Not recovering from fatigue |
|  | OR  (95% CI) | OR  (95% CI) | OR  (95% CI) | OR  (95% CI) | OR  (95% CI) | OR  (95% CI) | OR  (95% CI) |
| DRP<11 h | 0.85 | 1.02 | 1.36 | 1.31 | 1.09 | 0.94 | 0.95 |
|  | (0.69 - 1.04) | (0.81 - 1.28) | (1.00 - 1.86) | (0.89 - 1.95) | (0.77 - 1.55) | (0.74 - 1.21) | (0.74 - 1.21) |
| DRP≥11 h | 0.88 | 0.97 | 0.85 | 0.76 | 0.69* | 0.89 | 0.93 |
|  | (0.73 - 1.06) | (0.80 - 1.18) | (0.64 - 1.14) | (0.51 - 1.13) | (0.49 - 0.96) | (0.72 - 1.09) | (0.76 - 1.14) |
| Weekly working hours | 1.02*** | 0.99 | 1.01 | 1.01 | 1.03** | 1.02*** | 1.02*** |
|  | (1.01 - 1.04) | (0.98 - 1.00) | (0.99 - 1.03) | (0.98 - 1.03) | (1.01 - 1.05) | (1.01 - 1.04) | (1.01 - 1.04) |
| Women | 1.33** | 1.33** | 1.08 | 0.74 | 1.30 | 1.97*** | 1.79*** |
|  | (1.12 - 1.58) | (1.10 - 1.61) | (0.81 - 1.43) | (0.51 - 1.07) | (0.94 - 1.79) | (1.61 - 2.42) | (1.47 - 2.19) |
| Quantitative overload | 1.14*** | 1.04 | 1.11*** | 1.18*** | 1.04 | 1.22*** | 1.25*** |
|  | (1.10 - 1.19) | (1.00 - 1.08) | (1.05 - 1.18) | (1.08 - 1.28) | (0.97 - 1.12) | (1.17 - 1.28) | (1.19 - 1.30) |
| Observations | 4,130 | 4,138 | 4,139 | 4,138 | 4,138 | 4,136 | 4,121 |
| Pseudo R-squared | 0.0388 | 0.0375 | 0.0388 | 0.0549 | 0.0234 | 0.0575 | 0.0519 |
| Notes: *** p<0.001, ** p<0.01, * p<0.05. DRP: daily rest period. All models adjusted for age, education, marital status, family size, health behaviors, tenure, shift work, and occupation. | | | | | | | |
